# Towards a global arctic-alpine model for Near-infrared reflectance spectroscopy (NIRS) predictions of foliar nitrogen, phosphorus and carbon content

**DOI:** 10.1038/s41598-019-44558-9

**Published:** 2019-06-04

**Authors:** Francisco Javier Ancin Murguzur, Marjorie Bison, Adriaan Smis, Hanna Böhner, Eric Struyf, Patrick Meire, Kari Anne Bråthen

**Affiliations:** 1 Department of Arctic and Marine Biology, UiT Arctic University of Norway, N-9037 Tromsø, Norway; 2 Laboratoire d’Ecologie Alpine, Université de Savoie, 73376 Le Bourget du Lac, France; 3 Ecosystem Management Research Group, University of Antwerp, B-2610 Antwerp, Belgium

**Keywords:** Near-infrared spectroscopy, Biogeochemistry

## Abstract

Near-infrared spectroscopy (NIRS) is a high-throughput technology with potential to infer nitrogen (N), phosphorus (P) and carbon (C) content of all vascular plants based on empirical calibrations with chemical analysis, but is currently limited to the sample populations upon which it is based. Here we provide a first step towards a global arctic-alpine NIRS model of foliar N, P and C content. We found calibration models to perform well (R^2^_validation_ = 0.94 and RMSEP = 0.20% for N, R^2^_validation_ = 0.76 and RMSEP = 0.05% for P and R^2^_validation_ = 0.82 and RMSEP = 1.16% for C), integrating 97 species, nine functional groups, three levels of phenology, a range of habitats and two biogeographic regions (the Alps and Fennoscandia). Furthermore, when applied for predicting foliar N, P and C content in samples from a new biogeographic region (Svalbard), our arctic-alpine NIRS model performed well. The precision of the resulting NIRS method meet international requirements, indicating one NIRS measurement scan of a foliar sample will predict its N, P and C content with precision according to standard method performance. The modelling scripts for the prediction of foliar N, P and C content using NIRS along with the calibration models upon which the predictions are based are provided. The modelling scripts can be applied in other labs, and can easily be expanded with data from new biogeographic regions of interest, building the global arctic-alpine model.

## Introduction

The essential role of N and P in plants and ecosystem functioning has been emphasized over the last decades^1–9^. However, foliar N and P content are among the plant traits with the highest intraspecific variability^10^ and requires intense sampling. To enable further progress in our understanding of how and why N and P vary, and with what consequence, larger and larger sample sizes, encompassing interspecific and intraspecific variability at both spatial and temporal scales within and across ecosystems, are needed. However, efficient and low cost methodologies to meet these demands are largely missing and hence studies typically include a sub-optimal sample size^11,12^. That is, methodologies to assess plant nutrient content are characterized by high laboratory costs and destructive analytical methodologies such as the Kjeldahl digestion for N content^13,14^ and colorimetry for P content^15^. Costly and destructive analyses are among methodological shortcomings central to the ecological shortfalls^16^, and hence in order to move forward there is a demand for more cost-effective and non-destructive methods.

Near-infrared reflectance spectroscopy (NIRS), a well-established indirect measurement method for plant constituents routinely applied in agriculture^17^, holds the potential to overcome these limitations. NIRS is based on the light absorption of organic bonds in molecules (such as C-H, N-H, O-H) in the visible and near infra-red spectrum of light^18,19^. The combined absorptions at different wavelengths hold information about the content of the nutrients or constituent of interest^20^. The advantages of NIRS are manifold: because the analytical process largely can be omitted once calibration models are in place, processing costs can be reduced up to 80%^12,21^. Furthermore, the method is non-destructive and multiple constituents can be analysed simultaneously.

One of the challenges with NIRS methodology is that its application is limited to closed sample populations^11^. This means spectral characteristics of sample types not included in the calibration model may interfere with model predictions and cause spurious results. This limitation restrains the application potential of NIRS for ecological studies, because the range of multiple ecological contexts is not accounted for when developing population-specific calibration models. However, for foliar content of essential elements such as N and P, this interference is likely to be low. Organic molecules of plant leaves in which N and P is embedded (such as chlorophyll, amino acids, nucleic acids and phospholipids), are common among all terrestrial plants^22^ and are hence independent of ecological context. Studies on tree leaves support the potential for a global NIRS model for foliar N^23,24^ and foliar P^23^ as well as for foliar C^24^. Even studies on silicon, a non-essential element occurring in inorganic form in leaves of several functional types of vascular plants, support the potential for a global NIRS model^21^. Therefore, we hypothesised that NIR spectra can be used for modelling foliar N, P and C content across a range of functional types and ecological contexts and across a range of biogeographic regions.

The precision of NIRS calibrations for chemical constituents is dependent on the precision and bias of the analytical techniques from which the chemical constituents are retrieved and the NIR spectra are fitted^11^. Although within the acceptable range of precision requirements that apply to standard method performance for analytical methods^25^, any analytical technique imprecision reduces the fit between the actual constituent values and the NIR spectra^26^. Because precision requirements are lower for small contents^27^, the fit can be especially low for nutrients with small content. Furthermore, any bias, i.e. a systematic shift in measured quantity above or below the true content, will reduce the fit with NIRS derived spectra. Nevertheless, an applicable range of content should be applied in order to maximize method (calibration model) performance^25^. In addition, the magnitude of the imprecision can be reduced by using large sample sizes and thereby reduce the dependency on single, potential imprecise measurements.

In order to test the hypothesis that NIR spectra can be modelled for foliar N, P and C content across a range of functional types, ecological contexts and biogeographic regions, we included foliar samples of species belonging to nine functional groups, three phenological stages, a range of habitats and two different biogeographic regions. With this wide range of samples we also maximized the range of foliar N, P and C content, adhering to guidelines for how to develop optimally performing methods^25^. We developed NIRS calibration models and evaluated their capacity to accurately estimate foliar N, P and C content of a total of 552, 291 and 424 samples respectively. First, we evaluated the performance of calibration models based on biogeographically closed samples. Then we tested to what extent biographically distinct calibration models were transferable; We predicted Fennoscandian samples with calibration models based on samples from the Alps and *vice versa*. Finally, we assessed the performance of the arctic-alpine models incorporating samples from both biogeographic regions. For an assessment of the global potential of the arctic-alpine models, we tested model performances for samples from a new biogeographic region in addition to samples of a new functional group and a new phenological stage. We also evaluated the performance of the calibration models in light of precision requirements that apply to standard method performance for analytical methods.

## Results

### Foliar N, P and C content based on chemical analysis

The samples covered a large range of foliar N, P and C content (Table 1), and ranges from the Alps and Fennoscandia were largely overlapping. The total range of foliar content (in % dry weight) was 0.34 to 6.01% for N, 0.04 to 0.70% for P and 32.56 to 56.22% for C (Table 1) and extends the 2.5% and 97.5% quantile of the values in the TRY database encompassing several thousand species entries^28^. The functional types differed 2-3-fold in their average foliar N and P content. Legumes, forbs and deciduous trees had the largest foliar N content, and forbs, deciduous shrubs and horsetails the largest foliar P content. The foliar content of C was more similar among the functional types (Table 1).

**Table 1:** The mean and range of foliar N, P and C content (% dry weight) per functional groups per biogeographic region, the Alps (A) or Fennoscandia (F) and the arctic-alpine model, along with the number of species and the total sample size upon which the foliar content is assessed.

Functional group	Region	Nitrogen (N % dry weight)				Phosphorus (P % dry weight)				Carbon (C % dry weight)			
		No. species	No. samples	Mean	Range	No. species	No. samples	Mean	Range	No. species	No. samples	Mean	Range
Legumes	A	5	9	3.26	1.47–4.64	3	5	0.15	0.10–0.19	5	9	44.74	39.93–46.78
Forbs	A	47	159	2.95	0.34–5.63	34	66	0.26	0.04–0.70	47	138	44.74	33.21–50.90
	F	8	53	2.81	1.35–5.32	6	31	0.26	0.11–0.53	8	39	46.77	41.76–51.69
Grass	A	8	47	2.30	0.78–6.01	8	34	0.18	0.06–0.52	8	32	45.00	40.27–47.39
	F	9	114	1.75	1.02–3.75	8	65	0.17	0.07–0.56	9	77	45.93	43.17–48.03
Sedges/Rushes	A	2	11	1.56	1.31–2.16	2	6	0.12	0.08–0.17	2	11	45.33	43.65–46.87
	F	1	24	2.32	0.97–4.11	1	10	0.21	0.08–0.36	1	16	47.82	44.16–49.94
Horsetails	F	1	12	2.23	1.08–3.36	1	5	0.23	0.12–0.35	1	8	38.40	32.56–42.97
Deciduous shrubs	A	7	46	2.10	0.71–4.45	6	21	0.18	0.07–0.43	7	40	46.91	43.6–50.26
	F	3	17	2.31	1.33–4.02	3	16	0.32	0.11–0.63	3	11	50.75	48.02–53.26
Evergreen shrubs	A	6	27	1.12	0.68–2.44	6	11	0.08	0.04–0.21	6	25	50.65	45.82–53.29
	F	1	6	1.04	0.94–1.2	1	6	0.13	0.11–0.17	1	1	56.22	—
Deciduous trees	A	5	18	2.89	2.01–5.83	5	12	0.20	0.11–0.48	5	13	48.13	44.68–53.12
Evergreen trees	A	2	10	1.29	0.85–2.06	2	8	0.21	0.11–0.29	2	4	48.70	47.98–48.94
Overall	A	82	326	2.49	0.34–6.01	66	158	0.20	0.04–0.70	82	272	45.87	33.21–53.29
	F	23	226	2.11	0.94–5.32	20	133	0.21	0.07–0.63	23	152	46.37	32.56–56.22
Arctic-alpine model		97	552	2.33	0.34–6.01	79	291	0.21	0.04–0.64	96	424	46.05	32.56–56.22

### Method performances

The average relative standard deviation (RSD) for *within* laboratory precision of colorimetric measures of foliar P content was 6% (Table 2) (based on five replicates for each of three samples ranging from 0.13 to 0.23% P dry weight). In comparison, the average RSD for *within* laboratory precision of NIRS derived measures (based on three replicate scans for every sample in the arctic-alpine model), was 4.8% for P, 2.8% for N and 0.65% for C (Table 2). According to the precision requirements that apply to standard method performance for analytical methods^25^, these RSD values were marginally acceptable for the replicate measures of the colorimetric method and well within the accepted range for the replicate NIRS scans (Table 2).

**Table 2:** Results from tests of method precision. The relative standard deviation (RSD), also termed coefficient of variation, is a measurement of method precision advocated by the Guidelines for Standard Method Performance Requirements^25^.

Method	Measure	Replicates and Samples	Nitrogen (N)	Phosphorus (P)	Carbon (C)
Colorimetric measurements	Average RSD	Five measurements per sample		6%	
	Foliar content	Three samples		0.18%	
	RSD accepted			5.16%	
NIRS predicted measurements	Average RSD	Three scans per sample	2.8%	4.8%	0.65%
	Foliar content (From Table 1)	All samples	2.47%	0.21%	45.73%
	RSD accepted		3.48%	5.08%	2.25%

The foliar content is based on chemical analysis, and provides the basis for which the RSD accepted value is calculated.

**Figure 1:**
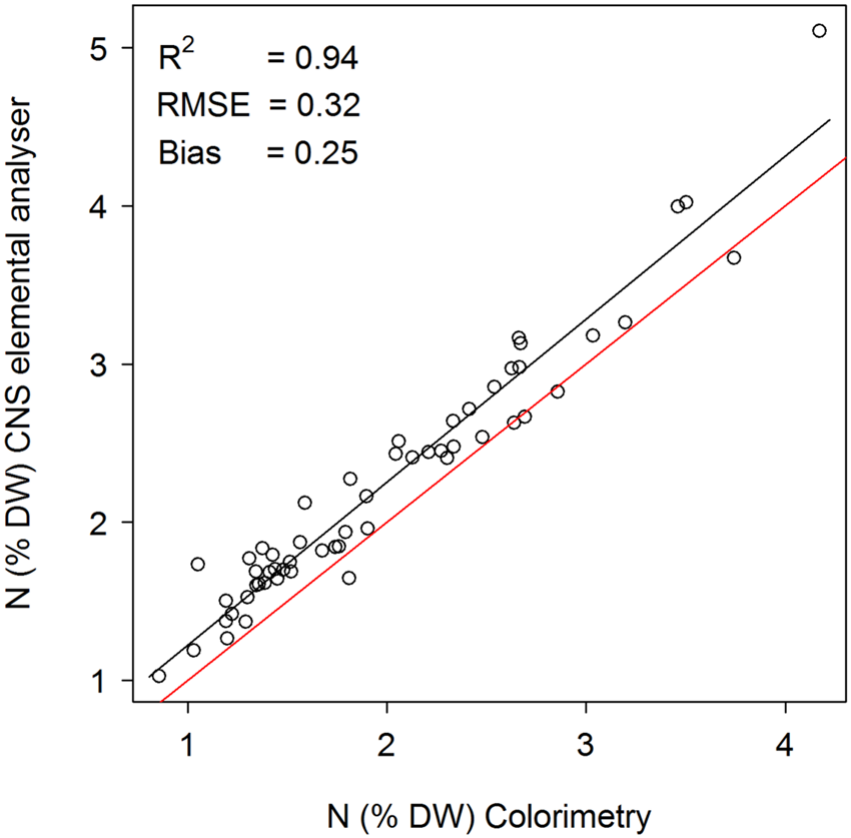
The relationship between N content (% dry weight) analysed using colorimetry and a CNS elemental analyser. Correlation coefficient (R^2^), root mean standard error (RMSE) and bias are presented. The red line shows the 1:1 relationship, and the black line shows the linear fit between the two methods.

The agreement *among* laboratories as estimated from the foliar N content of samples measured by both the colorimetric method and the CNS elemental analyser was R^2^ = 0.94 and with a RMSEP = 0.24, and showed a bias of approximately 0.15% N with the foliar N content measured by CNS to be higher (Fig. 1).

### NIRS calibration and validation

**Figure 2:**
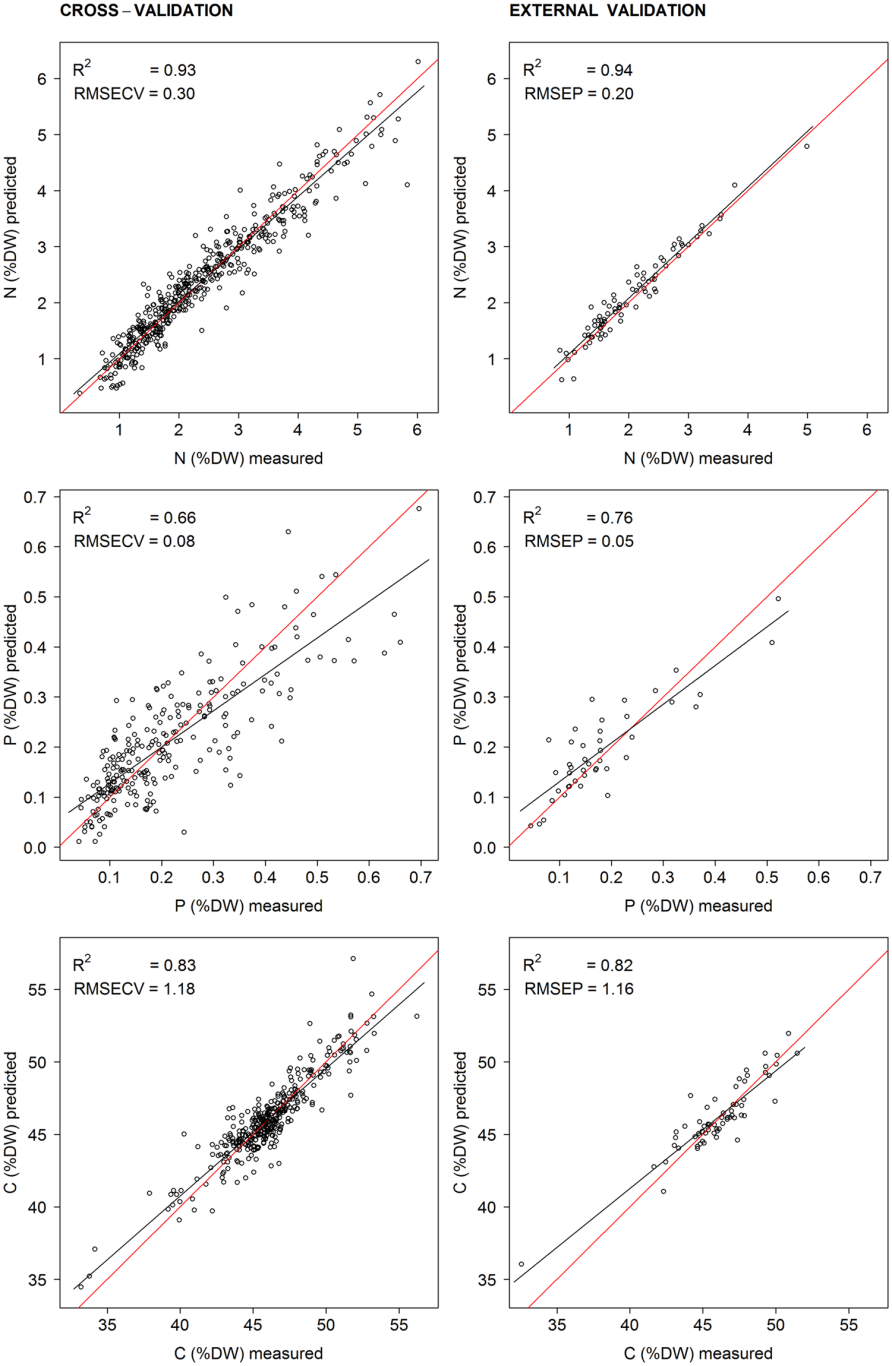
Cross-validation and external validation of the arctic-alpine NIRS calibration models in predicting laboratory measured content of foliar N, P and C (% dry weight). Each plot is accompanied by coefficient of determination (R^2^), root mean standard error of the cross validation (RMSECV) or external validation (RMSEP). The red line shows the 1:1 relationship and the black line shows the linear fit between the measured and predicted values. The list of species and their foliar N, P and C content upon which these models are based is provided in Table S1.

The biogeographic region specific calibration models showed a similar performance (Table 3). The best models were obtained for foliar N content (R^2^ = 0.94, RMSEP = 0.17 for Fennoscandia and R^2^ = 0.93, RMSEP = 0.27 for the Alps) and for foliar C content (R^2^ = 0.87, RMSEP = 1.16 for Fennoscandia and R^2^ = 0.89, RMSEP = 0.8 for the Alps) (Table 3). The models for foliar P content had reduced precision (R^2^ = 0.68, RMSEP = 0.07 for Fennoscandia and R^2^ = 0.70, RMSEP = 0.07 for the Alps) (Table 3). All arctic-alpine models were similar in performance to their region-specific counterparts (Table 3, Fig. 2), with slightly reduced, unchanged or slightly improved model parameters.

**Table 3:** Performance of region specific calibration models and arctic-alpine calibrations models for foliar N, P and C content (in % dry weight).

	Nitrogen (N)			Phosphorus (P)			Carbon (C)		
	The Alps	Fennoscandia	Arctic-alpine	The Alps	Fennoscandia	Arctic-alpine	The Alps	Fennoscandia	Arctic-alpine
***Cross-validation***									
k	20	18	17	6	10	13	21	16	15
R^2^ cval	0.96	0.96	0.93	0.70	0.68	0.66	0.88	0.83	0.83
RMSECV	0.24	0.16	0.30	0.07	0.07	0.08	1	1.08	1.18
***External validation***									
R^2^val	0.93	0.94	0.94	0.71	0.58	0.76	0.89	0.87	0.82
RMSEP	0.27	0.17	0.20	0.08	0.06	0.05	0.8	1.16	1.16
Bias	−0.03	−0.02	−0.08	−0.01	0.01	0.01	−0.04	−0.19	−0.13
Intercept	0.09	0.26	0.09	0.08	0.07	0.05	1.99	3.36	8.8
Slope	0.97	0.88	0.99	0.60	0.61	0.77	0.96	0.93	0.81

Model parameters are shown for two biogeographic region specific models and the arctic-alpine NIRS model including samples from both biogeographic regions, i.e. Fennoscandia and the Alps. Model parameters are presented for both cross-validation and external validation of the calibration models, including k = number of latent variables, R^2^cval = R^2^ for cross validation, RMSECV = Root Mean Standard Error of Cross Validation, R^2^val = R^2^ of the validation set, RMSEP = Root Mean Standard Error of the Prediction, Bias = mean error between estimated and measured values, Intercept and Slope of the linear fit.

When assessing the precision for each of the region-specific calibration models in predicting foliar N, P and C content in samples from the other region, both N calibration models performed well but both models had a considerable bias. Both the P and C calibration models had considerably lower precision in the predicted foliar P and C content of samples (Table 4).

**Table 4:** Performance of predictons of foliar N, P and C content (in % dry weight) using region specific calibration models.

Model is from	The Alps	Fennoscandia	The Alps	Fennoscandia	The Alps	Fennoscandia
Samples are from	Fennoscandia	The Alps	Fennoscandia	The Alps	Fennoscandia	The Alps
***Prediction***						
R^2^	0.86	0.88	0.56	0.37	0.66	0.70
RMSEP	0.28	0.38	0.14	0.13	1.19	1.36
Bias	0.42	0.57	0.12	0–0.01	2.05	2.43
Intercept	−0.19	0.54	0.01	0.08	18.02	−3.09
Slope	0.96	0.94	0.52	0.69	0.59	1.09

Calibration models from one region were used to predict content in foliar samples from the other region. Model parameters are k = number of latent variables, R^2^ = R^2^ of the sample set, RMSEP = Root Mean Standard Error of the Prediction, Bias = mean error between estimated and measured values, Intercept and Slope of the linear fit.

### Model performances for new sample types

**Figure 3:**
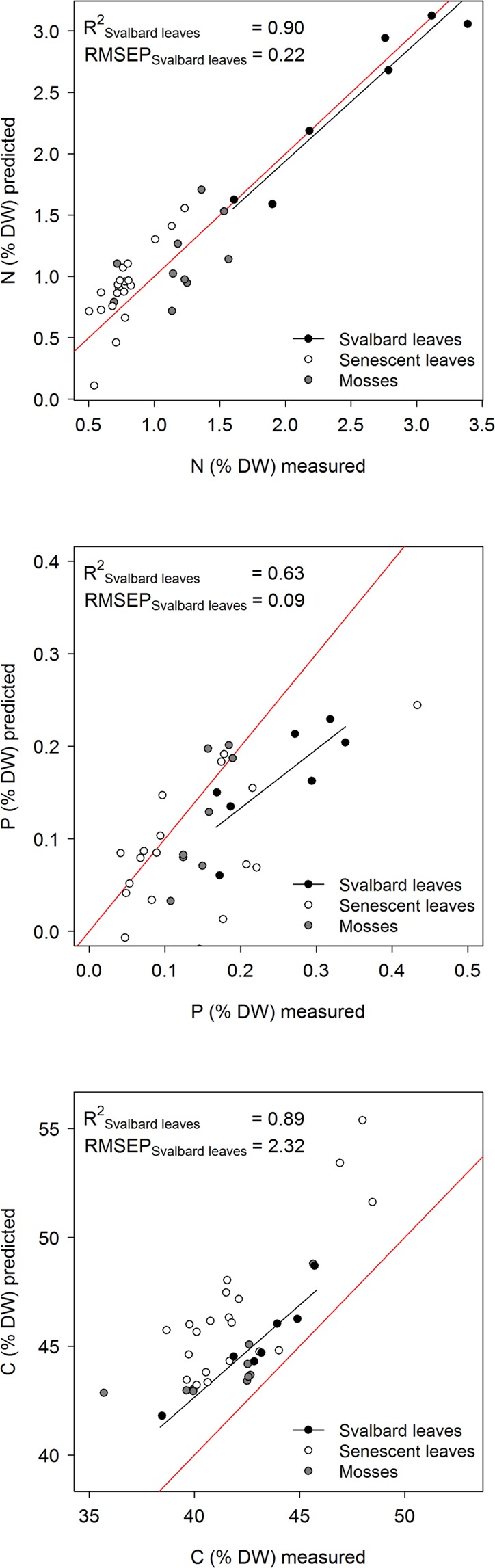
The relationship between N, P and C content of new sample types measured using chemical methods and predicted using the arctic-alpine NIRS calibration models. Each plot is accompanied by coefficient of determination (R^2^) and root mean standard error of prediction (RMSEP) for the relationship between predicted and measured foliar samples from Svalbard. The red line indicates the1:1 relationship. The list of species and their foliar N, P and C content is provided in Table S2.

Samples from the new biogeographic region, Svalbard, had average N, P and C contents similar to the samples used to develop the arctic-alpine models, but with more narrow ranges (Tables 1 and S2). The arctic-alpine models performed well when predicting the foliar N, P and C content of the Svalbard samples, despite a small sample size (n = 7) (Table S3, Fig. 3).

Both the senescent foliar samples and the moss samples had low average and narrow ranges of N and P contents in comparison to the samples used to develop the arctic-alpine models, whereas the average C content was similar (Tables 1 and S2). The arctic-alpine models performed less well for all these samples, especially the P model (Table S3).

The arctic-alpine calibration models were only slightly modified when incorporating the new sample types (Table S4), with all new samples blending in (Fig. S1).

## Discussion

Our results show that foliar N, P and C content can be measured by NIRS across a great variability of plant species and plant functional groups, providing a promising outlook for global arctic-alpine NIRS-based models. Our result is based on samples from 97 species belonging to a range of phenological stages and habitats, including variants of herbaceous and evergreen foliage. In total, the range of foliar nutrient content applied in this study corresponds to a ~18–fold difference in N content, a ~16-fold difference in P content and a ~2-fold difference in C-content, and encompassed the range of tree foliar content of N, P and C of that included in previous global models on tree species alone^23,24^. The cost efficiency of these global models opens avenues for incorporating foliar N, P and C in large scale ecological studies. This is strengthened by the fact that one scan of one sample provides N, P and C content and is non-destructive, causing scanned plant material to be available for further studies such as analysis on other constituents and follow-up ecological studies.

Our results showed that region specific models performed better with samples from the same region supporting the assumption that local models are good for predicting local samples and with a loss in precision when predicting outside the closed sample population^11^. However, our arctic-alpine models performed similar to the region-specific models indicating they overcome the limitations of transferability. The overall similarity in performance between the regional and the arctic-alpine models indicate the species pool differences between the two biogeographic regions were not interfering with the spectral properties associated to the foliar N, P and C content. Hence, our results suggest arctic-alpine models overcome limitations by regional models and make the prediction of foliar nutrient content across different biogeographic regions possible.

Our results also suggest that all our NIRS calibration models comply with the standards according to the guidelines for standard method performance requirements^25^, with RSD of models being within the accepted range of precision. Importantly, the accepted RSD range increases exponentially with smaller contents^27^. Because foliar P content is small in comparison to foliar N and C content, the accepted RSD of P is the largest. The lower performance of the foliar P content calibration model can thus be expected because it is trained against reference values with a lower precision^26^. This interpretation has support also from other studies where calibration models along with their validation models are better for foliar N content than for foliar P content despite similar sample sizes and ranges in N and P content^23,29^. Also, good performing N calibration models have been found in several other studies^23,24,29–31^. However, our foliar C calibration model, based on the largest content and hence the most precise measures, was still not the best performing model. In a previously published global model on N and C content for tree species, the best performing model was based on the largest range in content^24^. The discrepancies in model performance may thus also be due to differences in the range of N and C content: Also in our study the foliar N model was based on the largest range of content in samples. The range of C content included in the C model was less than 2-fold, significantly smaller than that of the N and P models. A wider range C content of samples will likely demand other tissues than leaves and thus, it is unlikely the calibration model of foliar C content will be improved much further. The arctic-alpine P model had approximately half the sample size to that of the N model and may improve with an increase in sample size, reducing dependencies on single imprecise measures. Hence, although our NIRS calibration models comply with the standards according to the guidelines for standard method performance requirements, expanding the N and P arctic-alpine models with more samples will likely both improve their performances and, if samples are from new biogeographic regions, build them towards global arctic-alpine models.

The calibration model on foliar N content was the best performing model in our study, yet its performance may be underestimated. The development of our NIRS calibration model for foliar N content was based on reference values from two different analysis methodologies, and has likely caused lower performance of the model^26^. The precision requirements that apply to standard method performance are stricter for within than between laboratories, with the accepted precision level within laboratories being 1/2 to 2/3 of that admitted among laboratories^25^. Accordingly, our comparison of foliar N content among laboratory measurements (which did not admit any calculation of RSD), showed a root mean square error (RMSE) and bias indicating a non-perfect fit. Interestingly, the fit between N content measured by the two chemical methods were in the order of that achieved for the N content predicted with our NIRS models (RMSE and RMSEP values provided in Fig. 1 and Table 3). Our results thus support the finding that NIRS calibration models can be as precise as the chemical analysis methods upon which the NIRS calibration models are based^26^.

The calibration models for foliar N, P and C content all performed well when tested on foliar samples from a new biogeographic region, supporting the outlook for global arctic-alpine models. However, when sample types of small contents not included in the original modelling (senescent leaves and mosses) were tested, the model performances declined, and especially so for mosses. Besides that mosses are non-vascular plants and hence structurally different from the functional types included in the original modelling, the reduced model performances is likely due to that senescent leaves and mosses both have small N and P content in comparison to green foliage of vascular plants. Method precision is expected to be lower with smaller content, and besides, the N and P content of senescent leaves and mosses were in the lower range of that covered by the models. However, although the arctic-alpine models performed poorly in differentiating content among samples of mosses and senescent leaves, the model predictions fell in the correct range of N and P content for these sample types. And when these sample types were included in the calibration models, they showed the same variation as with the original samples (Fig. S1) and the models were only slightly modified (Table S4). In summary, the models performed well for green foliar samples of a new biogeographic region whereas the models performed less well for other sample types not included in the original modeling.

Our study provides the first step towards global arctic-alpine NIRS calibration models for foliar N, P and C content. Importantly, and as demonstrated, our models can simply be assessed for their compatibility with new samples, or our models can be improved by adding new samples of new species and functional types, making the models even more independent of the origin of the samples. Furthermore, the raw spectral data upon which our calibration models are based, can be retrieved and modelled again with new statistical methods yet to be developed. We believe this study opens avenues for incorporating foliar N, P and C in large scale ecological studies, avenues likely to be even greater in the future.

## Methods

### Plant samples

The sampling was conducted in two biogeographic regions in Europe, in the Bauges Mountains in the French Alps and in Finnmark, the Norwegian part of Fennoscandia. The Bauges Mountains are a calcareous massif (altitude range 250–2217 m asl) characterized by a continental climate with an oceanic influence. Finnmark is the northernmost county in Norway, characterized by an undulating sandstone plateau of continental climate in its southern parts towards a more alpine landscape in coastal climate in its northern and western parts. The alpine tundra of the Bauges Massif and the sub-arctic tundra of Finnmark are biogeographic regions also in terms of wildlife and animal husbandry^32,33^.

We collected samples from a total of 97 different vascular plant species, with 82 species from the Alps and 23 species from Fennoscandia, and with eight species occurring in both regions (Table S1). The species belonged to at least nine different functional groups, i.e. legumes, other forbs, grasses, sedges and rushes, deciduous and evergreen shrubs, deciduous and evergreen trees. To maximize N, P and C content variability within species due to phenological changes^34^, sampling was conducted early, mid and late season in the summer. Moreover, to maximize N, P and C content variability both within and between species, sampling was conducted in a range of different habitats, including heath, scree, meadows, scrublands, grassland and megaphorbia in the Alps, and heath and grasslands along 14 different river catchments representing a set of different ecological contexts across northern Fennoscandia. In total 326 samples from the Alps and 226 samples from Fennoscandia were collected. Plant samples were stored in paper bags and air-dried in the field, and in the lab dried at 50 °C for 24 h and stored until sample preparation for scanning.

### Sample preparation

Plant samples were ground into fine powder using a ball mill (Mixer Mill, MM301; Retsch GmbH & Co. Haan, Germany) and pressed into tablets (Ø 16 mm, 1 mm thick) using a hydraulic press with 4 tons of pressure. This sample treatment created a homogeneous surface and reduced random light scattering^21^. Because water shows strong absorption patterns in the near infra-red region^35^ the tablets were oven dried for 2 h at 50 °C to remove any potential water films, after which samples were cooled to room temperature (approx. 20 °C) and stored in a desiccator until NIRS scans were taken.

### Spectral measurements

Each sample was scanned using a portable NIRS spectrometer (FieldSpec 3, Asd Inc., Boulder, Colorado). Spectra were recorded with monochromatic radiation in the wavelength range of 350–2500 nm with NIR, SWIR1 and SWIR2 sensors. The spectra were interpolated to 1 nm intervals based on recordings every 1.4 nm in the 350–1050 nm region and every 2 nm from 1050 to 2500 nm. Wavelength regions where the different sensors overlap (i.e. 350–380 nm, 760–840 nm, 1700–1800nm and 2450–2500 nm), were removed from the dataset due to potential inaccuracy in readings. Also the visible part of the spectrum (380–720 nm) was removed because this wavelength region has absorption features relevant for foliar traits^24^ that might emphasize leaf structural differences. Each final sample spectrum was the average of 3 replicate scans recorded as absorbance (log 1/R, where R = reflectance).

### Chemical analysis

C and N content (in % dry weight) of samples from the Alps (n = 272) were analysed using a CHN elemental analyser (Flash EA 1112, Thermo Electron Corporation). A subset of these samples with enough remaining material for further analyses (n = 104) were analysed for P content and an additional set of samples (n = 54) were analysed for both N and P content by colorimetry using a segmented flow analyser after chemical digestion^15^. One set of samples from Fennoscandia (n = 152) were analysed for their C and N content by a CNS elemental analyser (Flash 2000 Organic elemental analyser, Thermo Scientific, UK), one set (n = 59) were analysed for their P content and yet another set of samples (n = 74) were analysed for both N and P content, using the same colorimetric method as for the samples from the Alps. For all chemical analysis the recovery was at least 90% of Certified Reference Material (BCR-129 Institute for Reference Materials and Measurements at the European Commission Joint Research Centre).

### Assessment of method performance

For an assessment of method performance, we compared within and among laboratory derived N, P and C content for a subset of the samples following guidelines for precision requirements that apply to standard method performance for analytical methods^25^. Following these guidelines, method precision is estimated as relative standard deviation (RSD), also termed the coefficient of variation, and is calculated as the standard deviation of a set of replicate measurements, divided by their average and presented as a percentage (%).

First, for a RSD assessment *within* laboratory of chemical analysis sensu^25^, we measured the P content in five replicates for each of three samples from the Alps (samples for which we had enough material to do replicated measures). We also estimated *within* laboratory RSD of predictions based on the NIRS derived spectra. We predicted N, P and C content (see below) for each of the three replicate scans per sample separately. The within laboratory RSD was calculated as the average RSD across all samples. RSD values below or equal to RSD accepted values, calculated based on the formula RSD_accepted_ = 2 × content^−0.15^ (AOAC International 2016), were considered indicative of good method performance.

For a RSD assessment of N content measured *among* laboratories sensu^25^ along with an assessment of potential bias, some of the Fennoscandian samples (n = 56) were analysed by both the CNS elemental analyser and the colorimetric method. An assessment of bias requires a minimum of five replicate analyses of a Certified Reference Material^25^, which we did not have. We have nevertheless included a measure of bias by assessing the average difference in N content provided by the two methods of chemical analysis.

### NIRS calibration and validation

Spectral data transformations were applied during model development, with the use of centering, scaling, standard normal variate (SNV), smoothing based on moving averages, baseline corrections and 1^st^ and 2^nd^ order Savitzky-Golay derivatives^36,37^. A calibration and a validation subset of the data were created for each of the spectral data transformations. The calibration subsets were used to develop the models including internal cross-validation, whereas the final models were tested using the validation subsets (also termed external validation). The sample selection method for the two subsets was chosen so as to maximize model performance. The subsets were based on maximizing spectral variation using the Kennard-Stone algorithm^38^, with a ratio of calibration to validation sample sizes of 85:15. Spectral outliers were identified by means of Mahalanobis distances.

Each calibration model was developed using the partial least squares regression^39^, with a ten-fold cross-validation^40^ of the model to select the optimal model. The most parsimonious models were chosen based on an evaluation of the coefficient of determination (R^2^), the number of latent variables (k) and the root mean square of the error of the cross-validation of the calibration (RMSECV), which gives an assessment of the error between the predicted and the measured value. Finally, each calibration model was tested against its respective validation set (external validation). The coefficient of determination (R^2^), root mean standard error of prediction (RMSEP), bias (systematic error of the linear fit) and intercept and slope of the linear fit of the predictions were calculated to assess the robustness of the calibration.

Statistical analyses were all run in R 3.1.0 (R Development Core Team, 2014) using the partial least squares regression^39^ in the PLS package^41^ and first (1D) and second (2D) derivative treatments using the Prospectr package^37^.

### Region specific and arctic-alpine calibration models

First, we modelled biogeographic region-specific calibration models for foliar N, P and C content. Then we assessed the performance of each of these calibration models in predicting the foliar N, P and C content of samples from the other region. Finally, we combined the two spectral databases and developed arctic-alpine models for foliar C, N and P content.

### Model performance for new sample types

We assessed the performance of the arctic-alpine models in predicting N, P and C content of new sample types (Table S2). The new samples types were foliar samples of species from the Arctic (i.e. Svalbard as a new biogeographic region), senescent leaf samples from Fennoscandia (i.e. a new phenological stage) and moss samples (i.e. both a new functional group and non-vascular plant). The samples were processed and scanned as described above and the NIR spectra were applied for predicting foliar N, P and C content with the arctic-alpine models. The predicted values were compared to N, P and C content as retrieved from chemical analysis using a CNS elemental analyser for C content and colorimetry for N and P content (described above). Finally we incorporated the new samples into the arctic-alpine calibration models and assessed whether the different sample types altered their performances.

## Supplementary information

Supplementary Information

## Data Availability

Sample foliar N, P and C content and spectral information and R script-based calibration development and prediction models are available at UiT Open Research Data (https://opendata.uit.no) and directly following this link (10.18710/CXRCUW).
