# Supplementary material for: Towards a global arctic-alpine model for Near-infrared reflectance spectroscopy (NIRS) predictions of foliar nitrogen, phosphorus and carbon content
Source: Sci Rep. 2019 Jun 4;9:8259. doi: 10.1038/s41598-019-44558-9 (PMC6547662; doi:10.1038/s41598-019-44558-9)
Supplement: Supplementary file 1 — Supplementary Information [file 41598_2019_44558_MOESM1_ESM.pdf]

Supplementary Information for

Towards a global arctic-alpine model for Near-infrared reflectance spectroscopy (NIRS) predictions of foliar nitrogen, phosphorus and carbon content

Francisco Javier Ancin Murguzur[1], Marjorie Bison[2], Adriaan Smis[1,3], Hanna Böhner[1], Eric Struyf[3], Patrick Meire[3], Kari Anne Bråthen[1]*

[1] Department of Arctic and Marine Biology, UiT - The Arctic University of Norway, N-9037 Tromsø, Norway

[2] Laboratoire d'Ecologie Alpine, Université de Savoie, 73376 Le Bourget du Lac, France

[3] Ecosystem Management Research Group, University of Antwerp, B-2610 Antwerp, Belgium

* Corresponding author : kari.brathen@uit.no, tel +47 776 46629

Table S1. Foliar N, P and C content (% dry weight) of samples from a range of species and functional groups sampled in the Alps and in Fennoscandia. Species that are common to the Alps and Fennoscandia are marked with an asterisk (*).

Samples from the Alps

Functional group	Nitrogen (% dry weight)			Phosphorus (% dry weight)			Carbon (% dry weight)		
Species	No samples	Mean	Range	No samples	Mean	Range	No samples	Mean	Range
Forbs									
*Alchemilla alpina**	4	1.77	1.56 - 1.99	2	0.12	0.1 - 0.13	3	45.71	45.32 - 45.94
Alchemilla vulgaris	6	3.03	1.94 - 4.24	2	0.27	0.24 - 0.3	6	44.79	43.67 - 45.76
Arnica montana	3	1.66	1.02 - 2.53	1	0.32	-	3	43.61	42.98 - 44.49
Aruncus dioicus	3	2.76	2.08 - 3.21	1	0.41	-	3	46.06	45.93 - 46.23
Astrantia major	3	2.73	1.92 - 3.38	1	0.16	-	3	44.39	43.17 - 45.29
*Bistorta vivipara**	2	2.72	2.21 - 3.23	1	0.21	-	2	46.87	46.36- 47.38
Cardamine pentaphyllos	1	4.99	-				1	43.05	-
Carduus defloratus	3	3.13	1.6 - 5.24	1	0.12	-	3	41.94	39.74 - 43.89
Centaurea uniflora	1	2.28	2.28 - 2.28				1	44.31	-

Chaerophyllum hirsutum	5	3.69	2.77 - 4.69	1	0.41	-	5	44.04	43.17 - 44.99
Crocus vernus	1	4.19	-				1	45.84	-
Epilobium angustifolium	12	3.82	2.45 – 5.14	4	0.49	0.23 - 0.70	9	45.64	44.93 - 46.96
Filipendula ulmaria	2	2.69	2.34 - 3.05	1	0.15	-	2	44.70	44.51 - 44.88
Fragaria vesca	4	2.03	1.71 - 2.68	2	0.38	0.32 - 0.44	2	45.62	45.05 - 46.18
Galium odoratum	2	1.7	1.62 - 1.77	1	0.14	-	2	42.01	41.21 - 42.81
Gentiana purpurea	3	2.36	1.28 - 3.02	3	0.17	0.09 - 0.3	3	46.95	45.85 - 48.64
*Geranium sylvaticum**	7	2.425	0.93 - 3.76	6	0.21	0.08 - 0.37	3	46.36	45.81 - 46.88
Geum montanum	1	1.53	-				1	45.97	-
Globularia nudicaulis	3	1.08	0.89 - 1.39	2	0.05	0.04 - 0.06	3	46.40	44.33 - 48.4
Helianthemum nummularium	2	2.85	2.3 - 3.4	3	0.18	0.12 - 0.28	2	45.01	44.69 - 45.34
Heracleum sphondyllium	3	3.10	2.53 - 3.78	1	0.35	-	3	43.16	42.3 - 43.95
Hieracium murorum	3	1.91	1.76 – 2.08	3	0.22	0.17 - 0.27	2	43.59	43.13 - 44.04
Hypericum montanum	1	2.68	-				1	46.82	-
Hypericum richerii	2	2.25	1.61 - 2.88				2	46.24	45.08 - 47.4

Knautia arvense	6	3.34	2.63 - 3.68	1	0.32	-	6	46.34	45.1 - 47.17
Laserpitium latifolium	3	3.29	1.94 - 4.32	1	0.46	-	3	43.11	41.15 - 45.71
Leontodon hispidus	1	1.68	1.68 - 1.68			-	1	43.27	43.27 - 43.27
Mercurialis perennis	1	3.26	3.26 - 3.26	1	0.32	-	1	40.05	40.05 - 40.05
Origanum vulgare				1	0.04	-			
Phyteuma spicatum	2	3.83	2.28 - 5.37				2	43.63	42.62 - 44.63
Plantago atrata	2	2.50	2.33 - 2.66	1	0.13	-	2	44.14	43.25 - 45.03
Polygala chamaebuxus	1	1.30	-				1	50.90	-
Potentilla erecta	1	1.80	-				1	44.21	-
Pulsatilla alpina	3	2.41	1.29 - 3.78	1	0.34	-	3	43.65	40.96 - 47.07
Ranunculus tuberosus	15	2.94	2.05 - 4.68	3	0.32	0.22 - 0.51	12	43.04	41.64 - 45.2
Rhinanthus alectorolaphus	1	2.60	-				1	43.90	-
Rumex alpinus	12	4.89	3.85 - 5.4	4	0.49	0.35 - 0.65	9	47.44	45.82 - 49.16
Rumex arifolius	2	3.55	2.79 - 4.31				2	43.20	43.06 - 43.34
Salvia pratensis	1	2.50	-				1	44.96	-
Sanguisorba minor	1	3.92	-				1	44.08	-

Saxifraga rotundifolia	2	1.28	1.16 - 1.39	1	0.17	-	2	44.58	43.82 - 45.34
Sempervivum tectorum	2	0.60	0.34 - 0.86	2	0.06	0.04 - 0.07	2	33.49	33.21 - 33.77
Serratula tinctoria	2	2.09	1.85 - 2.34	2	0.11	0.09 - 0.14	2	45.10	45.04 - 45.16
Silene dioica	2	2.35	1.99 - 2.72	1	0.25		1	43.05	-
Taraxacum officinale	2	2.45	2.28 - 2.61	2	0.24	0.19 - 0.29	1	42.54	-
Urtica dioica	3	3.14	1.99 - 4.42	3	0.37	0.24 - 0.46	1	37.90	-
Valeriana montana	9	2.60	1.51 - 3.58	2	0.12	0.07 - 0.16	9	46.63	45.07 - 47.64
Veratrum album	8	3.91	2.8 - 4.89	1	0.23	-	8	45.32	44.22 - 46.11
Legumes									
Anthyllis vulneraria	2	2.14	1.47 - 2.82				2	40.38	39.93 - 40.83
Lathyrum pratensis	2	3.54	3.49 - 3.58	2	0.17	0.15 - 0.19	2	46.11	45.72 - 46.5
Onobrychis montana	2	3.09	2.93 - 3.26	2	0.13	0.1 - 0.15	2	46.35	46.25 - 46.46
Trifolium badium	1	3.42	-				1	43.61	-
Trifolium pratense	2	4.19	3.74 - 4.64	1	0.17	-	2	46.68	46.58 - 46.78
Grass									
Dactylis glomerata	9	3.71	2.25 - 6.01	6	0.31	0.18 - 0.52	7	45.58	44.48 - 47.39

*Deschampsia cespitosa**	8	2.42	1.61 - 4.64	6	0.19	0.01 - 0.34	5	44.02	40.27 - 45.71
*Festuca ovina**	5	1.37	1.21 - 1.47	4	0.11	0.09 - 0.13	3	45.87	44.91 - 46.46
Festuca rubra	1	3.69	-	1	0.27	-	1	45.40	-
Hordelymus europaeus	9	2.61	1.94 - 3.90	5	0.27	0.13 - 0.38	7	44.43	43.34 - 47.37
Melica uniflora	1	2.79	-	1	0.18	-	1	44.05	-
*Nardus stricta**	9	1.18	0.98 - 1.43	7	0.08	0.06 - 0.10	5	44.84	44 - 45.37
Sesleria caerulea	4	1.33	0.78 – 2.44	4	0.08	0.06 - 0.1	3	45.93	45.51 - 46.69
Sedges and Rushes									
Carex sempervirens	8	1.49	1.31 - 1.71	4	0.10	0.08 - 0.13	8	45.43	43.65 - 46.87
Luzula sylvatica	3	1.74	1.49 - 2.16	2	0.16	0.15 - 0.17	3	45.07	44.48 - 45.44
Deciduous shrubs									
Lonicera xylosteum	9	2.53	2.06 - 4.45	3	0.23	0.17 - 0.33	9	45.43	44.48 - 47
Rosa montana	7	2.28	1.64 - 3.74	2	0.28	0.15 - 0.41	7	45.91	43.6 - 47.6
Rubus fruticosus	11	2.01	1.39 - 3.15	4	0.19	0.13 - 0.32	9	45.44	44.21 - 46.11
*Salix sp.**	4	2.67	1.99 - 3.68	2	0.28	0.16 - 0.40	3	49.72	48.78 - 50.26
Sambucus nigra	1	3.79	-			-	1	44.70	-

*Vaccinium myrtillus**	5	1.24	0.71 - 1.75	3	0.09	0.07 - 0.11	4	49.96	49.55 - 50.18
Vaccinium uliginosum	9	1.66	1.14 - 2.20	5	0.10	0.08 - 0.14	7	49.08	48.23 - 50.03
Evergreen shrubs									
Arctostaphylos uva-ursi	11	0.87	0.68 - 1.37	5	0.07	0.04 - 0.09	9	51.12	48.9 - 53.29
Hedera helix	1	2.38	-	1	0.21	-	1	46.54	-
Juniperus communis	1	0.79	-	1	0.07	-	1	50.58	-
Rhododendron ferrugineum	11	1.12	0.9 - 1.35	2	0.05	-	11	51.48	49.58 - 52.09
Thymus serpyllum	2	2.12	1.8 - 2.44	1	0.1	-	2	45.84	45.82 - 45.86
Vaccinium vitis-idaea	1	0.87	-	1	0.05	-	1	51.18	-
Deciduous trees									
Acer pseudoplatanus	3	2.58	2.43 - 2.84	2	0.18	0.17 - 0.19	3	45.94	45.68 - 46.3
Alnus viridis	8	3.05	2.57 - 4.61	3	0.17	0.11 - 0.2	7	50.02	48.09 - 53.12
Fagus sylvatica	5	2.30	2.01 - 2.53	5	0.16	0.15 - 0.18	1	47.64	-
Fraxinus excelsior	1	2.48	-	1	0.21	-	1	44.68	-
Sorbus chamaemespilus	1	5.83	-	1	0.48	-	1	45.36	-

Evergreen trees									
Abies alba	5	1.24	0.85 - 2.06	3	0.23	0.20 - 0.28	3	48.93	48.93 - 48.94
Picea abies	5	1.33	1.00 - 1.88	5	0.20	0.11 - 0.29	1	47.98	-

Samples from Fennoscandia

Functional group	Nitrogen (% on dry weight)			Phosphorus (% on dry weight)			Carbon (% on dry weight)		
Species	No samples	Mean	Range	No samples	Mean	Range	No samples	Mean	Range
Forbs									
*Alchemilla alpina**	22	2.63	1.35 - 4.2	17	0.24	0.12 - 0.42	16	46.97	45.38 - 51.69
*Bistorta vivipara**	4	3.50	3.01 - 4.1				4	47.75	46.76 - 49.05
*Geranium sylvaticum**	8	2.14	1.4 - 3.99	5	0.21	0.11 - 0.45	5	47.54	46.25 - 48.35
Ranunculus sp.	2	2.92	2.49 - 3.34	1	0.36	-	1	46.19	-
Rumex acetosa	4	3.61	1.92 - 5.32	1	0.53	-	3	46.97	46.66 - 47.49
Solidago virgaurea	6	3.56	2.12 - 5.13	3	0.30	0.21 - 0.45	4	47.48	46.67 - 49.13
Trollius europaeus	5	2.43	1.51 - 3.48	4	0.32	0.14 - 0.51	4	43.51	41.76 - 45.36

Viola sp.	2	3.00	2.9 - 3.09				2	46.41	46.33 - 46.48
Grass									
Agrostis tenuis	3	2.37	1.95 - 2.72	4	0.19	0.12 - 0.23	1	45.97	-
Alopecurus pratensis	2	2.09	1.53 - 2.64	1	0.07	-	1	47.87	-
Anthoxanthum nipponicum	10	1.89	1.3 - 2.54	5	0.20	0.1 - 0.26	8	46.33	45.12 - 47.66
Avenella flexuosa	23	1.72	1.05 - 2.48	10	0.24	0.09 - 0.36	17	46.75	45.49 - 47.85
Calamagrostis phragmitoides	16	1.86	1.19 - 3.19	15	0.15	0.09 - 0.34	7	46.16	44.46 - 47.7
*Deschampsia cespitosa**	30	1.69	1.08 - 2.18	10	0.11	0.08 - 0.17	28	45.07	43.17 - 47.18
*Festuca ovina**	1	2.47	-				1	46.23	-
*Nardus stricta**	19	1.56	1.25 - 2.27	11	0.14	0.10 - 0.20	12	45.94	43.98 - 47.51
Phleum alpinum	10	1.69	1.02 - 3.75	9	0.19	0.07 - 0.56	2	47.46	46.88 - 48.03
Sedges and Rushes									
Carex sp.	24	2.32	0.97 - 4.11	10	0.21	0.08 - 0.36	16	47.82	44.16 - 49.94
Horsetails									
Equisetum sp.	12	2.23	1.08 - 3.36	5	0.23	0.12 - 0.35	8	38.4	32.56 - 42.97

Deciduous shrubs									
Betula nana	4	1.71	1.53 - 2.16	4	0.25	0.19 - 0.35	2	53.03	52.8 - 53.26
*Salix sp.**	12	2.58	1.33 - 4.02	11	0.37	0.11 - 0.63	8	50.08	48.02 - 52.78
*Vaccinium myrtillus**	1	1.64	-	1	0.12	-	1	51.58	-
Evergreen shrubs									
Empetrum nigrum	6	1.04	0.94 - 1.2	6	0.13	0.11 - 0.17	1	56.22	56.22 - 56.22

Table S2. Foliar N, P and C content (% dry weight) of new sample types representing a new biogeographic region (Svalbard), a new phenological state (senescent leaves from Fennoscandia) and a new functional group (mosses from Svalbard). Species that are common to the ones in the arctic-alpine model are marked with an asterisk (*).

Foliar samples from Svalbard

Functional group		Nitrogen (% dry weight)		Phosphorus (% dry weight)		Carbon (% dry weight)	
Species	No samples	Mean	Range	Mean	Range	Mean	Range
Forbs							
*Bistorta vivipara**	1	3.39	-	0.34	-	44.91	-
Grass							
Alopecurus borealis	1	2.18	-	0.17	-	41.88	-
Dupontia fisheri	2	2.20	1.61 - 2.79	0.23	0.17 - 0.29	43.02	42.85 - 43.19
Sedges							
Eriophorum scheuchzeri	1	3.12	-	0.32	-	43.94	-

Horsetails							
Equisetum sp.*	1	2.76	-	0.27	-	38.48	-
Deciduous shrubs							
Salix polaris	1	1.9	-	0.19	-	45.71	-
All Svalbard species	7	2.59	1.61 - 3.39	0.25	0.17 - 0.34	42.99	38.48 - 45.71
**Senescent leaves**							
Forbs							
Taraxacum sp.	1	1.13	-	0.13	-	38.69	-
Alchemilla sp.	2	0.7	0.60 - 0.80	0.14	0.07 - 0.22	39.93	39.74 - 40.12
Geum sp.	2	0.77	0.72 - 0.82	0.1	0.09 - 0.10	40.82	40.12 - 41.52
*Achillea millefolium**	1	0.78	-	0.18	-	41.57	-
*Cicerbita alpina**	1	0.81	-	0.43	-	39.78	-

*Geranium sylvaticum**	2	0.73	0.72 - 0.73	0.34	0.22 - 0.45	42.61	42.12 - 43.10
*Filipendula ulmaria**	1	0.68	-	0.08	-	44.03	-
*Solidago virgaurea**	1	0.55	-	0.05	-	40.77	-
Grass							
Poaceae sp.	3	0.93	0.76 - 1.23	0.07	0.04 - 0.08	40.65	39.64 - 41.68
*Deschampsia cespitosa**	1	0.6	-	0.04	-	40.56	-
Rushes							
Luzula sp.	2	0.88	0.74 - 1.10	0.18	0.17 - 0.18	41.71	41.64 - 41.78
Deciduous shrubs							
*Betula pubescens**	1	0.71	-	0.1	-	48.47	-
*Vaccinium myrtillus**	2	0.64	0.50 - 0.77	0.07	0.05 - 0.09	47.47	46.93 - 48.00
All species with senescent leaves	20	0.76	0.50 - 1.23	0.15	0.04 - 0.45	42.08	38.69 - 48.47

Bryophytes

Mosses							
Tomenthypnum nitens	3	1.04	0.72 - 1.25	0.16	0.12 - 0.21	41.72	39.96 - 42.68
Sanionia uncinata	3	1.36	1.18 - 1.36	0.18	0.16 - 0.19	38.43	35.70 - 39.95
Aulacomnium sp.	2	0.92	0.70 - 1.14	0.12	0.11 - 0.15	42.56	42.54 - 42.58
Polytrichum sp.	2	1.4	1.23 - 1.57	0.14	0.12 - 0.15	44.14	42.61 - 45.66
All moss species	10	1.18	0.70 - 1.57	0.15	0.11 - 0.21	41.71	35.70 - 45.66

Figure S1. Cross-validation and external validation of the arctic-alpine NIRS calibration models including foliar samples from a new biogeographic region (Svalbard), from a new phenological stage (senescent leaves) and a new functional group (moss samples), in predicting laboratory measured content of foliar N, P and C (% dry weight). Each plot is accompanied by coefficient of determination (R^2), root mean standard error of the cross validation (RMSECV) or external validation (RMSEP). The red line shows the 1:1 relationship and the black line shows the linear fit between the measured and predicted values. The list of the new species and their foliar N, P and C content upon which these extended arctic-alpine models are based is provided in Table S2.

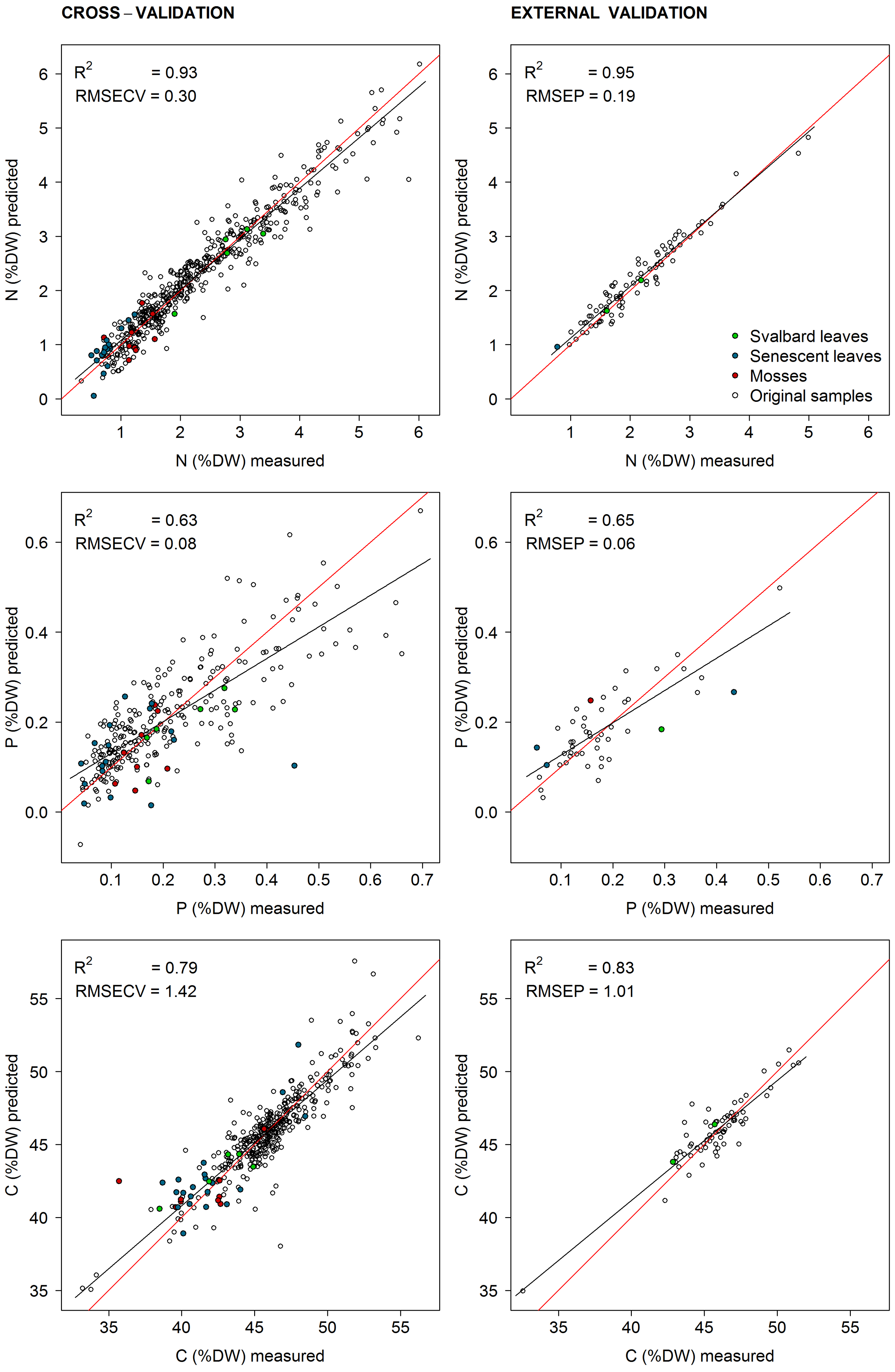

CROSS – VALIDATION
EXTERNAL VALIDATION
R2 = 0.93
RMSECV = 0.30
R2 = 0.95
RMSEP = 0.19
Svalbard leaves
Senescent leaves
Mosses
Original samples
N (%DW) predicted
N (%DW) measured
R2 = 0.63
RMSECV = 0.08
R2 = 0.65
RMSEP = 0.06
P (%DW) predicted
P (%DW) measured
R2 = 0.79
RMSECV = 1.42
R2 = 0.83
RMSEP = 1.01
C (%DW) predicted
C (%DW) measured

Table S3. Performance of the NIRS arctic-alpine calibration models in predicting foliar N, P and C content (in % dry weight) in samples from a new biogeographic region (Svalbard), a new phenological stage (senecent leaves) and a new functional group (mosses). Model parameters are R^2 = R^2 of the sample set, RMSEP = Root Mean Standard Error of the Prediction, Bias = mean error between estimated and measured values and Intercept and Slope of the linear fit.

	Nitrogen (N)			Phosphorus (P)			Carbon (C)		
	Svalbard leaves	Senescent leaves	Mosses	Svalbard leaves	Senescent leaves	Mosses	Svalbard leaves	Senescent leaves	Mosses
R^2	0.90	0.62	0.23	0.63	0.44	0.24	0.89	0.69	0.53
p-value	<0.01	<0.01	0.16	0.03	<0.01	0.15	<0.01	<0.01	0.02
RMSEP	0.22	0.32	0.30	0.09	0.08	0.08	2.32	4.87	3.19
Bias	0.10	-0.22	0.07	0.09	0.04	0.05	-2.20	-4.52	-2.67
Intercept	-0.02	-0.21	0.47	0.01	0.02	-0.07	8.78	4.77	23.87
Slope	0.97	1.55	0.53	0.64	0.52	1.14	0.85	0.99	0.49

Table S4. Performance of the arctic-alpine calibration models for foliar N, P and C content (in % dry weight) extended with additional samples from a new biogeographic region (Svalbard), a new phenological stage (senecent leaves) and a new functional group (mosses). Model parameters are presented for both cross-validation and external validation of the calibration models, including k = number of latent variables, R^2cval = R^2 for cross validation, RMSECV = Root Mean Standard Error of Cross Validation, R^2val = R^2 of the validation set, RMSEP = Root Mean Standard Error of the Prediction, Bias = mean error between estimated and measured values and Intercept and Slope of the linear fit.

	Nitrogen (N)			Phosphorus (P)			Carbon (C)		
	Svalbard leaves	Senescent leaves	Mosses	Svalbard leaves	Senescent leaves	Mosses	Svalbard leaves	Senescent leaves	Mosses
Cross-validation									
k	17	17	17	13	13	13	15	15	15
R^2 cval	0.93	0.93	0.93	0.63	0.62	0.66	0.81	0.80	0.81
RMSECV	0.30	0.29	0.30	0.08	0.08	0.07	1.20	1.30	1.10
External validation									
R^2val	0.94	0.95	0.93	0.76	0.67	0.66	0.82	0.87	0.84
RMSEP	0.20	0.21	0.23	0.06	0.06	0.07	1.2	1.0	1.1
Bias	-0.05	-0.04	-0.07	-0.01	-0.01	<0.01	-0.14	-0.17	-0.28

Intercept	0.13	0.05	0.25	0.07	0.07	0.07	9.6	6.7	8.2
Slope	0.96	0.99	0.93	0.70	0.72	0.68	0.79	0.86	0.83
